# Research on the Influence Mechanisms of the Affective and Cognitive Self-Esteem

**DOI:** 10.3390/ijerph192013232

**Published:** 2022-10-14

**Authors:** Shufang Yang, Mingyao Zhang

**Affiliations:** 1 Department of Industrial Engineering, Tsinghua University, Beijing 100084, China; 2 Antai College of Economics and Management, Shanghai Jiao Tong University, Shanghai 200030, China; 3 School of Physical Education and Sports Science, Lingnan Normal University, Zhanjiang 524048, China

**Keywords:** social media usage, loneliness, older adults, cognitive self-esteem, affective self-esteem, objective social isolation

## Abstract

Most prior studies examined the direct relation between social media usage and loneliness. This study tries to reveal the covert mechanisms involved in how different types of SMU affect older adults’ loneliness, which has rarely been an object of attention in the prior literature. A partial least squares structural equation modeling (PLS-SEM) method was used to analyze the data collected from 466 older adults in a field study. This research divided self-esteem into two dimensions: affective self-esteem (AE) and cognitive self-esteem (CE). The study found that changes in CE only stemmed from functional SMU (FSMU), rather than active SMU (ASMU) and passive SMU (PSMU). ASMU and PSMU had a significant effect on FSMU. CE had a significant effect on loneliness. Objective social isolation (OSI) had a positive relationship with loneliness. Moreover, PSMU, FSMU, and CE had a significant effect on ASMU, CE, and AE, respectively. For older adults, the feeling of connecting with others was more valuable than acquiring specific outcomes. The mediation test results showed that FSMU could play a completely mediating role in the relationship between ASMU and PSMU, as well as that between ASMU and CE. CE significantly mediated the relationship between FSMU and loneliness. Finally, the total effect sizes of ASMU and PSMU on FSMU were significant, and those of CE on older adults’ loneliness and AE were significant, while the total effect of AE on older adults’ loneliness was non-significant. AE moderated the relationship between PSMU and OSI, so PSMU was related to higher OSI only for users experiencing high AE. These findings offer a guide for the use of social media to conduct future loneliness interventions for older adults.

## 1. Introduction

Research has demonstrated that older people experience loneliness more often than young groups do [1,2]. Loneliness refers to a negative emotion experienced when the quality of social relationships is insufficient [3]. There was evidence showing that loneliness is associated with cognitive decline, dementia risk, early mortality risk, and cardiovascular disease for older adults [4]. Living with feelings of loneliness has implications for quality of life, physical and mental health, and mortality [1].

As social media usage [5] has grown exponentially throughout the last decade, older people have also become important participants. Social media has turned into a place for social activities related to conducting loneliness interventions for older adults [6]. Indeed, prior studies have revealed that SMU impacts older adults in many ways, such as depression, mental health, and loneliness [7,8,9,10]. On the one hand, loneliness can drive SMU [11,12,13,14,15,16,17]. On the other hand, SMU has an impact on increasing or decreasing loneliness [13,18,19,20,21,22,23,24]. Compared with their attention toward younger age groups, these studies paid little attention to older adults [25,26,27]. Going beyond the direct association between SMU and loneliness, the covert mechanism connecting the two should be identified, especially for older adults. For example, shopping and paying on social media can decrease cognitive self-esteem, which, in turn, decreases loneliness. In addition, classifying different types of SMU helps with this issue (e.g., active and passive use) [28,29,30,31,32]. To our knowledge, no study has simultaneously investigated how different types of SMU (i.e., active, passive, or functional use) affect loneliness in older adults.

Based on a field study in China, 466 older adults were interviewed. A PLS-SEM method was used to examine how these types of SMU affect older adults’ loneliness. The cognitive self-esteem, affective self-esteem, and objective social isolation of older adults were also included. The research results revealed that SMU had a very different impact on loneliness for older adults than it does for young adults. Since preventing loneliness is more important than curing it, our study offers a guide for the use of social media to conduct future loneliness interventions for older adults [3].

### 1.1. Types of SMU

This typology is seemingly increasingly used in the literature. Gerson and his colleagues [31] have tried to develop a professional scale for this typology. In fact, active SMU can be classified into two sub-types (e.g., interactive and non-interactive types). This typology is understood according to the information process of older adults. Active SMU refers to information generation, while passive SMU refers to information consumption. However, there are activities on SM that do not (or only slightly) involve processes of emotional information, such as payment. We call these activities as functional SMU. Active and passive SMU refer to activities on SM that involve emotional information processes. 

While many studies have measured overall SMU, the most studied taxonomy of SMU is the framework that includes the active and passive types of usage [33]. Different social media activities may have distinguishing effects. Burke and her colleagues divided these activities into directed communication, broadcasting, and passive consumption [34,35]. Then, researchers theoretically developed these terms into active Facebook use and passive Facebook use [31,32]. According to the prior literature, we defined (a) passive SMU as using social media to consume information from others, (b) active SMU as using social media to exchange information with others, and (c) functional SMU as using social media to complete tasks in life with non-emotional involvement in information of others [28,30,31,32].

### 1.2. Social Media and Loneliness

Loneliness refers to the feelings associated with the absence of an intimate attachment or feelings of emptiness or abandonment [36]. Feeling lonely is not just emotionally painful, but literally deleterious to human health [3]. Loneliness occurs when one’s intimate and social needs are not adequately met. Social media have been identified as affording particular qualities that provide people with both transient and enduring feelings of connectedness [37]. As evidence, Hajek and König found that daily social media users reported lower loneliness scores compared with those with less frequent or no use [26]. A higher degree of SMU also resulted in users’ feeling that they are in the same community as others. The degree of salience—thereby closely imitating real-life social interactions—was effective in attenuating loneliness [22]. 

In terms of active SMU, users interact with their friends on social media and exchange their information through self-disclosure. Passive SMU permits users to observe overflowing information about others’ life events. Nonetheless, users do not become involved in others’ information when engaging in functional SMU. Additionally, older adults lack the ability to recognize cognitive adjustments, so they tend to trust information from others on social media [38]. Compared with younger people, lonely older adults tend to avoid engaging in social media due to their poor social abilities [39]. Accordingly, we propose the following hypothesis:

**Hypothesis** **H1a:** *ASMU directly increases older adults’ FSMU.*

**Hypothesis** **H1(b/c):** *PSMU decreases older adults’ (b) ASMU and (c) FSMU.*

**Hypothesis** **H1d:** *PSMU indirectly decreases older adults’ FSMU, mediated by an increase in ASMU.*

### 1.3. Objective Social Isolation

Social isolation refers to an objective state of lack of social contact [40]. Objective social isolation means that an individual lacks social contact in real life. While social isolation is one of the determinants of loneliness, people can feel lonely when not socially isolated [41]. Older adults with depression often have uncontrollable conflicts with their peers stemming from the pain of diseases and affective disorders, all of which can lead to social isolation [42]. Although OSI and loneliness are closely related, socially isolated older adults may report a low level of loneliness [41]. In addition, older adults with high OSI may have a strong motivation to use social media [26]. Social media use was positively correlated with mental health and negatively correlated with social isolation [43]. However, inappropriate use of social media was associated with perceived levels of social isolation [44]. Accordingly, we posited the following hypotheses:

**Hypothesis** **H2a:** *ASMU directly decreases older adults’ OSI.*

**Hypothesis** **H2(b/c):** *(b) PSMU and (c) FSMU directly increase older adults’ OSI.*

**Hypothesis** **H3:** *AE directly decreases older adults’ OSI.*

**Hypothesis** **H4:** *OSI directly increases older adults’ loneliness.*

### 1.4. Self-Esteem

Self-esteem mainly refers to the subjective evaluation of an individual’s own value, role, and attitude [45]. Low self-esteem is linked to poor mental health [46]. For example, low self-esteem can lead to apathy, isolation, and passivity [47]. Explicit self-esteem was negatively correlated with depressive symptoms, suicidal ideation, and loneliness [48]. Social media use has a positive impact on self-esteem [49], and it has been found that social media participation is positively correlated with group self-esteem [50]. Instagram and Facebook are regarded as active social media types, and users post positive content to increase the positive responses of subscribers, thereby enhancing their self-esteem [51]. More active TV watching is associated with lower social contact and self-esteem [52]. Passive Facebook use affected women’s internalization of societal beauty ideals, which were inversely correlated with women’s satisfaction with their bodies and self-esteem [53]. It was found that loneliness was negatively correlated with self-esteem and positively correlated with smartphone addiction, while self-esteem was negatively correlated with smartphone addiction [54]. In addition, due to the extensive existence of peer pressure and conformity, appropriate consumption at the right time is very important for self-esteem, social acceptance, and maintenance of social relations of specific groups. Some studies have suggested that the self-esteem of some groups has been commercialized, and there are many adverse consequences of failing to “keep up” with consumption trends, such as social exclusion, negative peer evaluation, and low self-esteem; this requires the strengthening of people’s correct understanding of self-esteem [55]. Moreover, level A of theoretical analysis at the RSES level specifies that the behavioral pattern of subjects in self-evaluations can be divided into cognitive and affective elements [56]. However, no studies have incorporated affective self-esteem and cognitive self-esteem into the framework of active, passive, and functional social media use based on objective social isolation theory. Thus, we proposed the following hypotheses:

**Hypothesis** **H5(a/b):** *ASMU directly increases older adults’ (a) AE and (b) CE.*

**Hypothesis** **H5c:** *ASMU indirectly decreases older adults’ CE, mediated by an increase in FSMU.*

**Hypothesis** **H6(a/b):** *PSMU decreases older adults’ (a) AE and (b) CE.*

**Hypothesis** **H7a:** *FSMU directly increases older adults’ AE.*

**Hypothesis** **H7b:** *FSMU directly decreases older adults’ CE.*

**Hypothesis** **H7c:** *FSMU indirectly decreases older adults’ AE, mediated by an increase in CE.*

**Hypothesis** **H7d:** *FSMU indirectly increases older adults’ loneliness, mediated by an increase in CE.*

**Hypothesis** **H8:** *CE directly increases older adults’ AE.*

**Hypothesis** **H9(a/b):** *(a) AE and (b) CE directly decrease older adults’ loneliness.*

**Hypothesis** **H9c:** *PSMU indirectly increases older adults’ CE, mediated by an increase in FSMU.*

**Hypothesis** **H10:** *For older adults, AE will enhance the effects that PSMU has on OSI.*

## 2. Methods

### 2.1. Participants and Procedure

The study began with a pilot study involving 32 older adults who lived in a care home. Some questions were originally written in English and then translated into and back-translated from Chinese by two of the researchers who were fluent in both languages. The results demonstrated sufficient validity and reliability. After that, a field study was conducted in another pension institution (hereafter, “the institution”) from May to July in 2019. The institution operated 8 locations located in Anhui Province, China. Since there were well-structured questions in this research, the caretakers were trained to interview the older adults for research efficiency. In order to reduce bias, all eight of the locations took part in the study. A total of 466 older adults were interviewed, of which 52.1 percent (N = 243) reported experience with SMU. The numbers of participants in the different locations ranged from 43 to 70. With regard to age (N = 460; M = 69.84; SD = 8.47), 94.5% were older than 60 years. Participants had predominantly low levels of education (82.7% with less than a high school degree) and were predominantly female (N = 456; 62.5%) and seemingly healthy (94.2%). Additionally, the main social media applications that participants adopted included WeChat, QQ, Weibo, and Toutiao.

### 2.2. Measures

Type of SMU: We adapted validated scales from prior studies [28,30,31,32,57] to measure passive and active SMU. Four and three items were used to evaluate passive and active SMU, respectively. The measurement for functional SMU was based on two additional items. Participants needed to evaluate how often they engaged in a number of activities while using any social media site, such as “Browsing content posted by friends”, “Commenting”, and “Shopping on social media”. The cutoff points were based on six-point Likert scales anchored at 1 (‘‘never’’) and 6 (‘‘several times one day’’).

Objective social isolation: A measure of objective social isolation from family and friends was used [40]. The frequency of contact with family members (friends) was measured by the question, “How often do you see, write or talk on the telephone with relatives (friends) who do not live with you?”. The cutoff points were based on seven-point Likert scales anchored at 1 (‘‘every day’’) and 7 (‘‘never’’).

Six items modified by Rosenberg were used to evaluate older adults’ self-esteem with respect to the institution [58,59]. The affective self-esteem measure was composed of three items that were related to the perceived affection of the older adults, and cognitive self-esteem measure was composed of three items that were related to the perceived cognition of the older adults. 

### 2.3. Statistical Techniques

This study adopted partial least squares structural equation modeling (PLS-SEM) as a statistical method. The SPSS24 and SmartPLS3 software was used to analyze the responses. Only 201 records (83%) were analyzed with PLS-SEM, since 42 of the social-media-using participants provided incomplete data. The PLS-SEM method was recommended for testing these complex hypotheses for the following reasons: (a) Our hypotheses formed a complex path model with latent variables that had many indicators [60]; (b) the size of our sample was small relatively for SEM analysis [61]; (c) older adults who lived in the same location may have been related to each other such that the distribution of our sample would not have met the requirements of the parametric factor-based SEM approach [62].

## 3. Results

Common method bias became a potential risk to the PLS-SEM analysis because we adopted the same interview procedure and scales for the participants [63]. Multiple methods were utilized to alleviate and assess the common method bias. As a remedy, the measures across different constructs eliminated common scale properties by changing the response formats across sections [64]. As a statistical assessment, Harman’s single-factor test showed that a single factor only explained 11% of the variance, which indicated a very low likelihood of the threat of common method bias [65].

The results of the PLS-SEM analysis firstly offered a reliable and valid measurement model that provided support for the measurement quality. Each of the constructs used within the PLS-SEM analysis was measured with a reflective indicator. Therefore, the indicator reliability, internal consistency reliability, convergent validity, and discriminant validity were evaluated. CFA loadings above 0.7 can indicate the indicator reliability. Some items of cognitive self-esteem and affective self-esteem were deleted because of their low CFA loadings. Cronbach’s Alpha, RHO_A, and composite reliability [48] values were examined to assess the internal consistency reliability, with all of them having a criterion value above 0.6 [66,67]. Convergent validity was assessed with the average variance extracted (AVE) with a criterion value above 0.5. The discriminant validity assessment involved analyzing the heterotrait–monotrait (HTMT) ratio of the correlations with a criterion value below 0.85 [68]. Table 1 and Table 2 display the reliability and validity of the measurement models after modification.

**Figure 1:**
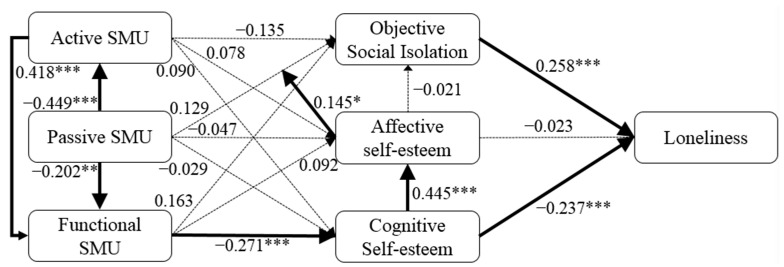
PLS-SEM results for the relationship between SMU patterns and older adults’ loneliness. Notes: SMU, social media usage; thick solid lines indicate significant effects, and thin dashed lines indicate insignificant effects; significance was assessed with the bootstrapping method (N = 2000); * *p* < 0.05, ** *p* < 0.01, and *** *p* < 0.001.

The structural model of the PLS-SEM analysis for the testing of the hypotheses is displayed in Figure 1. All VIF values among the constructs were below 5, which indicates that the collinearity was not serious. The results revealed that there was a unique pattern of relationships between SMU and loneliness for older adults with respect to that for younger age groups.

In terms of the antecedents of FSMU, ASMU (b = 0.418, t = 5.643, *p* < 0.001) and PSMU (b = −0.202, t = 3.120, *p* < 0.01) had a significant effect on FSMU, providing support for H1a and H1c. CE (b = −0.237, t = 3.501, *p* < 0.001) had a significant effect on loneliness. That is, H9b was supported. Moreover, OSI had a positive relationship with loneliness (b = 0.258, t = 3.320, *p* < 0.001), which was consistent with the findings of previous studies [41]. That is, H4 was supported. PSMU, FSMU, and CE had significant effects on ASMU (b = −0.449, t = 10.000, *p* < 0.001), CE (b = −0.271, t = 3.672, *p* < 0.001), and AE (b = 0.445, t = 5.349, *p* < 0.001), respectively. That is, H1b, H7b, and H8 were supported. For older adults, AE enhanced the effects that PSMU had on OSI (b = 0.145, t = 2.084, *p* < 0.05). Thus, H10 was supported.

The SmartPLS 3.3.3 software provides researchers with algorithms that can be used to acquire specific indirect effects in order to test mediation hypotheses [69]. The results of the mediation test showed that FSMU could play a mediating role in the relationships of ASMU (b = −0.113, t = 3.147, *p* < 0.01) and PSMU (b = 0.055, t = 2.248, *p* < 0.05) with CE. That is, H5c and H9c were supported. ASMU significantly mediated the relationship between PSMU and FSMU (b = −0.188, t = 4.668, *p* < 0.001). CE significantly mediated the relationship between FSMU and AE (b = −0.120, t = 3.106, *p* < 0.01). Moreover, CE significantly mediated the relationship between FSMU and loneliness (b = 0.064, t = 2.364, *p* < 0.05). That is, H1d, H7c, and H7d were supported, whereas the others were not. Finally, the results also showed that the total effect size of ASMU on FSMU was 0.418 (t = 5.643, *p* < 0.001), the total effect size of PSMU on FSMU was −0.389 (t = 7.239, *p* < 0.001), the total effect size of FSMU on CE was −0.271 (t = 3.672, *p* < 0.001), the total effect size of CE on AE was 0.445 (t = 5.349, *p* < 0.001), and the total effect size of CE on older adults’ loneliness was −0.249 (t = 4.203, *p* < 0.001), while the total effect size of AE (t = 0.402) on older adults’ loneliness was non-significant.

## 4. Discussion and Conclusions

From a motivation perspective, while alleviating loneliness is a motivator for young adults to use social media [16,17], it does not drive older adults. This result also reminds us that when examining the impact of SMU on older adults, we are studying a group of older adults who are less lonely.

Furthermore, our results revealed that SMU has a more limited impact on the loneliness of older adults than it does on the loneliness of young adults. Consistently with a prior study [32], changes in cognitive self-esteem only stem from functional SMU rather than from active and passive SMU. This implies that only more consumption activities on social media can result in cognitive self-esteem. Unlike active and functional SMU, passive SMU is mainly driven by browsing the information of others, leading one to face an enormous amount of information about others [29,70]. In terms of the antecedents of functional SMU, active SMU and passive SMU have a significant effect on functional SMU. Cognitive self-esteem has a significant effect on loneliness. Moreover, often, if older adults realize that their life is more isolated than those of others on social media, their lack of offline social connections will be more fatal. In addition, different types of SMU have an indirect impact on affective self-esteem and objective social isolation. 

Objective social isolation has a positive relationship with loneliness, which is consistent with the findings of previous studies [41]. Moreover, passive SMU, functional SMU, and cognitive self-esteem have a significant effect on active SMU, cognitive self-esteem, and affective self-esteem, respectively. For older adults, the feeling of connecting with others is more valuable than acquiring specific outcomes.

Moreover, the results of the mediation test show that functional SMU could play a completely mediating role in the relationships of active SMU and passive SMU with cognitive self-esteem. This implies that improving the functional SMU of older adults can reduce the impact of active SMU on their cognitive self-esteem and increase the impact of passive SMU on their cognitive self-esteem. In this sense, for older adults who actively and passively use social media, in order to change their cognitive self-esteem, they should be encouraged to engage in activities of consumption on social media. Cognitive self-esteem significantly mediates the relationship between functional SMU and loneliness. This suggests that in order to alleviate older adults’ loneliness through cognitive self-esteem, the first step should be to improve their concept of consumption. Finally, the results also showed that the total effect sizes of active SMU and passive SMU on functional SMU were significant; those of cognitive self-esteem on older adults’ loneliness and on affective self-esteem were also significant, while the total effect size of affective self-esteem on older adults’ loneliness was non-significant.This study explores the underlying mechanisms underlying different types of self-esteem. It reveals the internal relationship between cognitive self-esteem, affective self-esteem and mental health of the elderly, and provides theoretical support for the development of different types of self-esteem. This study provides a reference for elderly care institutions and government departments to develop mental and physical health management strategies for the elderly. Social media technology plays an important role in promoting the development of healthy aging and provides convenience for the elderly. Reasonable use of social media is conducive to alleviating the loneliness of the elderly and improving their quality of life. 

## 5. Limitations

Although this study has made some progress, there are still some limitations. First, although a sufficiently representative sample was obtained in this study, the survey’s objects mainly focused on the elderly in smart elderly care institutions, and the scope of the research had certain limitations. Second, due to the limitations of human resources and other factors, this study only collected data at a certain time point. Future research can increase the number of samples, consider longitudinal data factors, and collect data from different community pension institutions so as to make the study more extensive and representative. Third, this study may have overlooked other factors regarding the effects of social media use on loneliness in older adults. In addition, future research could consider other contributing factors.

## Figures and Tables

**Table 1:** Reliability and convergent validity.

Cronbach’s Alpha		RHO_A	CR	AVE
ASMU	0.918	0.918	0.960	0.924
PSMU	0.901	0.907	0.931	0.771
FSMU	0.623	0.637	0.840	0.725
OSI	0.774	0.777	0.899	0.816
CE	0.623	0.652	0.802	0.581
AE	0.648	0.650	0.810	0.588
LN	0.823	0.866	0.893	0.737

Note: ASMU, active SMU; PSMU, passive SMU; FSMU, functional SMU; CE, cognitive self-esteem; AE, affective self-esteem; OSI, objective social isolation; LN, loneliness.

**Table 2:** HTMT ratio of the correlations.

	ASMU	PSMU	FSMU	OSI	CE	AE
PSMU	0.493					
FSMU	0.677	0.496				
OSI	0.168	0.144	0.218			
CE	0.149	0.170	0.358	0.118		
AE	0.169	0.134	0.084	0.126	0.675	
LN	0.106	0.198	0.175	0.330	0.337	0.163

Note: ASMU, active SMU; PSMU, passive SMU; FSMU, functional SMU; CE, cognitive self-esteem; AE, affective self-esteem; OSI, objective social isolation; LN, loneliness.

## Data Availability

The data used in this study can be requested from the authors.
